# Relationships among Trust in Messages, Risk Perception, and Risk Reduction Preferences Based upon Avian Influenza in Taiwan 

**DOI:** 10.3390/ijerph9082742

**Published:** 2012-08-02

**Authors:** David Fang, Chen-Ling Fang, Bi-Kun Tsai, Li-Chi Lan, Wen-Shan Hsu

**Affiliations:** 1 Graduate Institute of Tourism and Health Science, National Taipei University of Nursing and Health Science, 365 Ming-te Road, Peitou District, Taipei 11281, Taiwan; Email: davidfang@ntunhs.edu.tw; 2 Department of Finance and Cooperative Management, National Taipei University, 151 University Road, San Shia District, New Taipei 23741, Taiwan; Email: faling@mail.ntpu.edu.tw (C.-L.F.); e73199@hotmail.com (W.-S.H.); 3 Graduate Institute of Bio-Industry Management, National Chung Hsing University, 250 Guoguang Road, South District, Taichun 40227, Taiwan; Email: pktsai@dragon.nchu.edu.tw

**Keywords:** avian influenza, trust in message, risk perception, risk reduction preference

## Abstract

Improvements in communications technology enable consumers to receive information through diverse channels. In the case of avian influenza, information repeated by the mass media socially amplifies the consumer awareness of risks. Facing indeterminate risks, consumers may feel anxious and increase their risk perception. When consumers trust the information published by the media, their uncertainty toward avian influenza may decrease. Consumers might take some actions to reduce risk. Therefore, this study focuses on relationships among trust in messages, risk perception and risk reduction preferences. This study administered 525 random samples and consumer survey questionnaires in different city of Taiwan in 2007. Through statistical analysis, the results demonstrate: (1) the higher the trust consumers have in messages about avian influenza, the lower their risk perceptions are; (2) the higher the consumers’ risk perceptions are and, therefore, the higher their desired level of risk reductive, the more likely they are to accept risk reduction strategies; (3) consumer attributes such as age, education level, and marital status correlate with significant differences in risk perception and risk reduction preferences acceptance. Gender has significant differences only in risk reduction preferences and not in risk perception.

## 1. Introduction

Avian influenza outbreaks can be divided into two categories: poultry infections and human infections. Although it spread only among poultry in Taiwan in 2004, the overall culling of chickens that occurred in several counties had previously prejudiced the Taiwanese against consuming chicken-related products.

Through the mass media transmission of information, the fear of chicken product consumption heightened the public’s risk perception. Risks can be amplified or attenuated by its social amplification. Kasperson and Kasperson used the signal amplification effect from communication theory to explain the spread of risks and indicated different sources of risk messages that amplified risk perception by transmission to the populace through multiple referral mechanisms and the interaction of repeated feedback [1]. Therefore, the process of information delivery may cause signal amplification, which influences consumers’ risk perception. Hence, media reporting, risk perception, and public reaction can affect consumers’ attitudes toward risk events. Risk events such as mad-cow disease and genetically-modified foods have demonstrated the socially-amplified-risk effect [2].

From the intensive media reporting about avian influenza and the risk-amplification effect, consumers feel anxious when facing uncertainty. It has been found that trust can reduce consumer uncertainty and trust is an effective mechanism for simplifying the risky situations in our daily lives [3]. Other studies indicate that in people concerned about food safety, risk perception comes from related information on risk management strategies [4,5]. Thus, because consumers develop strategies to reduce risks so that they can be more confident while facing unexpected situations, we should study more thoroughly consumer’s risk perception and reduction behavior intended to reduce their risks. For example, if consumers try to reduce risk rather than stop purchasing perceived high-risk items, they may take actions such as choosing reputable brands and shops, searching for related safety food information, and avoiding unsafe products.

When consumers believe the avian influenza information from the media, it may affect their risk perception and lower their risk tolerance. Therefore, this research examines the connection among consumers’ trust in messages, risk perception, and preference for risk reduction behavior. The purposes of the research are as follows:

1. To study how trust in messages affects risk perception when consumers receive avian influenza information from mass media.
2. To analyze the path how trust in messages affects risk perception and risk reduction preferences.
3. To find the individual demographic attributes impact trust in message, risk perception and level of risk reduction preference if the disease spreads.

## 2. Literature Review

### 2.1. Trust in Messages

In general, information recipients were affected by a wide range of messages, and organized their attitudes toward the information, including the confidentiality of information. In the next section, we will discuss *argument quality* and *source credibility* separately, then, we will take the two information characteristics as our measure dimensions.

Information quality is also called argument quality. In persuasive communication, argument quality is always an important concept while discussing information content or information structure. Most of the discussions focus on whether the information content is persuasive. Normally, when the information recipients perceive the information including rational methods and supporting proofs, as a strong argument, that information is more likely to be persuasive than a weak argument with unconvincing proof [6].

Petty and Cacioppo have thoroughly examined argument quality by using the so-called elaboration likelihood model (ELM) [7]. They divided information quality into two categories according to recipient’s predisposition: strong message and weak message. They defined a strong message as one which usually caused the research subjects’ preferred thoughts, while a weak message was defined as one which caused undesired thoughts [8,9]. In addition, a strong message as well proved by facts or statistical methods and a weak message as providing a low level of credibility [10]. Based on these studies, high-quality arguments cause the intended attitudes or thoughts in the recipients, while low-quality arguments cause the opposite or unintended attitudes or thoughts in the recipients.

Source credibility generally refers to positive characteristics of information providers, which influence the audience’s acceptance level. In many studies, we find that information recipients decide the source credibility on the basis of certain criteria. Source credibility is partly based on the public’s attitude toward the information source, trust, or skepticism, and partly upon the public’s interactions with that source [11]. In general, the recipient’s response was directly related to his/her perception of the media’s attributes. Therefore, although reporting same issues, media with different attributes may have different credibility levels. 

This study organizes source credibility dimensions in Table 1 on the basis of Ohanian’s study and other researchers’ recent arguments [12]. Although there are different opinions on source credibility dimensions, we used expertise and reliability as our source credibility variables because they are mentioned most frequently (as Table 1). Our definition is as follows [12,13,14,15,16,17,18,19,20,21,22,23,24].

1. Expertise: The information disseminator has expert knowledge and provides persuasive arguments.
2. Reliability: The information recipient’s degree of trust in information advocated by the disseminator.

These two dimensions (argument quality and source credibility) were commonly applied in previous research models. In past decades, most scholars focused on consumers’ change or formation of attitude when they are stimulated. Scholars suggested the ELM to describe how recipients manage information differently depending upon the content, their personal traits, and the situation in which they receive messages [25]. The process of management can be divided into two routes: the central route and the peripheral route.

**Table 1:** Dimensions of source credibility.

	Dimensions of source credibility												
Scholars (year)	Expertise	reliability	Dynamism	Attractiveness	Ability	Trustworthiness	Sociability	Objectiveness	Affinity/Accessibility/Agreeableness	Safety	Qualification	Care	Values
Bowers and Phillips (1967) [17]		★			★								
Whitehead (1968) [23]		★	★		★			★					
Berlo, Lemert and Mertz (1969) [16]			★							★	★		
Applbaum and Anatol (1972) [14]	★	★	★					★					
Simpson and Kahler (1980) [22]	★		★			★	★						
DeSarbo and Harshman (1985) [18]	★	★		★					★				
Wynn (1987) [24]	★		★			★	★						
Ohanian (1990) [12]	★	★		★									
Johnson (1999) [36]					★							★	★
Lafferty and Goldsmith (1999) [20]	★	★											
Belch and Belch (2001) [15]	★	★											
Kiecker and Cowles (2002) [19]	★	★		★									
Pornpitakpan (2003) [21]	★	★		★									
total	10	10	5	4	3	2	2	2	2	1	1	1	1

According to Petty and Cacioppo’s illustrations of the ELM, persuasion is completed through the central route when the subject has the ability and motivation to deal with the information. At the time, argument quality is an important variable of persuasion in the central route, when recipients notice the persuasive tactics in the information. Petty and Cacioppo suggested that if the information contains favorable arguments, the influence of messages could increase accordingly; otherwise, the information might be rejected. In addition, high-quality argument information stimulates the audience to think about it. On the other hand, if the subject lacks the ability and motive to deal with the information, persuasion is completed through the peripheral route. The peripheral route is a shortcut to making decisions, in which people are affected only by some peripheral cue such as emotion, attraction, source credibility, and type of message. Overall, people’s subjective judgments of source credibility play an important role in their response to the information. Researchers have suggested that when recipients do not understand the content of information, their degree of acceptance influenced by source credibility [26,27,28,29]. Consequently, this study will apply the central and peripheral routes of the ELM and use both argument quality and source credibility to measure information trustworthiness.

### 2.2. Trust in Messages and Risk Perception

Risk perception refers to people’s judgment about the severity of negative results, which becomes a mark or symbol probability. Such judgments are affected by personal attributes, experiences, information, ability to deal with information, importance of the events, voluntary action, and ability to control situations [30]. Scholars showed that risk perception can be affected by the way it is presented [31]. Let us investigate the effect of trust in the risk messages on risk perception. If consumers trust the information provided by the government or media and base their risk perceptions upon them, we can prove that trust is one of the significant factors influencing risk perception.

In many foreign studies, we observed that the trust in the government and industry is an important influential factor in risk perception and risk acceptance [32,33,34,35,36,37,38]. Many experimental proofs have shown that people’s trust in institutions dramatically affected risk perception and risk acceptance [39,40]. In technology risk studies, experimental results indicate that risk perception changes according to people’s previous experience in accepting information, especially the level of trust in the government and media.

General consumers obtain information from diverse channels such as mass media, institutions, government departments, and individual sources such as one’s experiences, relatives, friends, experts, and local media (newspaper, magazine, TV, broadcasting). The information source’s reputation plays a key role in risk information communication. A source’s lack of public trust will devalue its information and diminish consumers’ risk perception.

Many experimental results prove that relationships exist between risk perception and information trust. When consumers receive messages from media, the level of information trust is affected by many factors including professional knowledge, information source expertise, and frequency of message reception.

### 2.3. Risk Perception and Risk Reduction Preferences

Kahneman and Tversky’s study showed that people tend to take a risk when they are in the beneficial range of exposure; when they are in the loss range, they avoid risks [41]. To minimize the potential risk, people followed strategies such as obtaining the favorable product information through formal or informal sources, through buying products or brands having high-quality reputations, or purchasing the same products repeatedly (brand loyalty) in order to lower risks.

Roselius used 11 useful strategies to lower risks, including endorsement, brand loyalty, major brand image, private testing, store image, free sample, money-back guarantee, government testing, shopping, expensive model, and word of mouth [42]. He found that consumers with higher perceived risks exhibit more intention to use risk-reduction strategies than do consumers with lower perceived risks. In addition, five risk reduction strategies were prioritized according to the consumers’ responses as follows: product quality, product information, post-purchase control, place of purchase, and price [4]. Yeung and Yee also proved that the level of perceived risk correlates positively by using risk reduction strategies [43]. The preceding discussion shows that the possibility that the consumers will take risk reduction actions and will look for different risk reduction strategies dependent upon their level of risk perception increases with an increase in their risk perception.

Several related studies suggest that different levels of risk perception can result from various consumer attributes, which may lead to different levels of risk reduction strategies. Consumers’ risk perception was varies significantly according to the attributes such as demographic factors, behavior factors, and psychological factors [44]. The demographic factors include gender, age, job, monthly income, education level, marital status, and family life cycle. The following studies have proven that demographic factors influenced individuals’ responses to different types of risks. For example, gender does not have a significant influence on the public’s risk perception of avian influenza in another avian influenza study [45].

## 3. Methods

### 3.1. Research Framework and Assumptions

**Figure 1:**
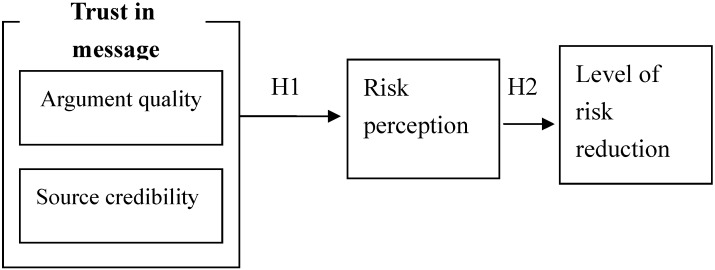
Framework of this research.

This study focused on consumers’ risk perception after they received avian influenza information and then that information’s effect on their level of risk reduction preference. According to previous studies and consumers’ choices of higher risk acceptance newspapers and magazines, we constructed the framework of this study in Figure 1.

The following research hypothesis were proposed as the foundation for the research framework:

H1: Consumers’ trust in messages has no significant influence on their risk perception ofavian influenza.
H2: Consumers’ risk perception does not significantly influence their level of risk reduction preference.

### 3.2. Subjects and the Questionnaire

The subjects were general consumers and a probability sampling technique was used for this study to generalize findings to a large population. Participants were chosen using simple random sampling by the proportion of the population of each city. This study gave out questionnaires by street survey to participants who were distributed randomly in northern, central, southern, and eastern regions of Taiwan from September to October 2007. We gave 100 questionnaires for a pretest and revised the questionnaire after collecting the pretest results. We received 525 questionnaires, of which 516 were valid. We chose one out of every five participants on the street and helped them to fill out the questionnaires personally. Thus, the response rate was 98.3%.

### 3.3. Questionnaire Design

This study provided reports about avian influenza from the Bureau of Animal and Plant Health Inspection and Quarantine for the target population to read before they completed the questionnaires [46]. The dimensions of the questionnaire design are illustrated as below.

**Table 2:** The measure items of argument quality.

Dimension	Item Description	
Argument quality	1.	The messages provided by the mass media can increase my understanding of the avian influenza.
	2.	The messages of the avian influenza provided by the mass media are quite valuable to me.
	3.	The messages of the avian influenza provided by the mass media are very helpful for me.

**Table 3:** The measure items of source credibility.

Dimension	Item Description	
Source credibility	1.	I often browse the mass media (newspapers, television) published reports, such as avian influenza. They are quite worthy of my trust.
	2.	I often browse the mass media (newspapers, television) published reports, such as avian influenza. They are quite reliable.
	3.	I often browse the mass media (newspapers, television) published reports, such as avian influenza. They are very professional.
	4.	In my opinion, the mass media (such as newspapers, television) that I often viewed is quite informative media.

#### 3.3.1. Trust in Messages—Argument Quality and Source Credibility

There are many studies providing methods for measuring the relationships between these two variables. In this study, we took Bhattacherjee and Sanford’s research variables and used a five-point Likert scale as our measurement, with 1 = “strongly disagree”, 3 = “neutral”, and 5 = “strongly agree” [29]. Therefore, the subjects’ trust in the information given in the questionnaire’s messages about avian influenza increases with an increase in the scores given by them. The questionnaire is shown in Table 2 and Table 3.

#### 3.3.2. Risk Perception

In this study, risk perception is defined as the estimation held by consumers about the chance of occurrence of a risk or about the extent, magnitude, and timing of their effects after got the messages of avian flu. This section of items follows the information on avian influenza from the Bureau of Animal and Plant Health Inspection and Quarantine. We used a five-point Likert scale as our measurement, with 1 = “strongly disagree”, 3 = “neutral”, and 5 = “strongly agree”. Therefore, the risk perception of avian influenza of the subjects increased with an increase in the scores given by them. The questionnaire is shown in Table 4.

**Table 4:** The measure items of risk perception.

Dimension	Item Description	
Risk perception	1.	The risk of the avian influenza strikes will be high.
	2.	The farmers of chicken and duck will get the avian influenza virus with very high degree.
	3.	The harmful levels of the avian influenza strikes to my family will be great.
	4.	The avian influenza viruses in Taiwan will have a significant impact on Taiwan’s economy.
	5.	The human-to-human transmission of the avian influenza will have a significant economic impact in Taiwan.

#### 3.3.3. Level of Risk Reduction Preference

Various risk reduction strategies are used in different studies. This study used risk reduction strategies of Yeung and Morris and Yeung and Yee to establish five types of strategies: product quality, product information, post-purchase control, place of purchase, and price [4,43]. Each category included two to three strategies, so we edited some items. From the result of the pretest, the variable price was neither unreliable nor invalid. The questionnaires also used a five-point Likert scale as our measurement, with the subjects’ higher scores indicating their higher level of risk reduction preferences for avian influenza. The questionnaire is shown in Table 5.

**Table 5:** The measure items of level of risk reduction preference.

Dimension		Item Description
Level of risk reduction preference	1.	To find the previous purchase of the stores or street vendors to buy.
	2.	To the well-known stores, such as large supermarkets to buy.
	3.	We will buy in those stores which will guarantee your money back if you are not satisfied with the quality.
	4.	Buy the chicken products with the brand.
	5.	Buy the chicken products with the brand which friends suggested.
	6.	Buy the chicken products which chicken authoritative experts suggested.
	7.	Purchase chicken products with the origin of information.

### 3.4. Reliability and Validity

Cronbach’s α is frequently used to evaluate the internal consistency of measuring tools. In this study, Cronbach’s α of dimension reliability examinees are exceeded 0.7, and were well within the commonly accepted range of reliability [47]. The validity is 0.928, which suggested that the questionnaire performed well on validity and had high consistency and stability of responses. 

Next, we examined construction validity through exploratory factor analysis to ensure that there are significant measurable relationships existing between the variables in each dimension. This analysis showed that each item fits well under pre-assumed dimensions and that there are significant measurable relationships existing between the variables and the concepts, which proved that the questionnaire possessed suitable validity. As Table 6 shows, all average variance and extracted and composite reliability values in this study significantly exceed 0.87. Convergent validity can be determined by reviewing the average variance extracted (AVE) and the composite reliability (CR) for each construct. In this study, these values indicate that the convergent validity in this study is acceptable.

**Table 6:** The analysis of reliability and validity among trust in messages, risk perception, and level of risk reduction preferences.

Variables	Cronbach’s α	Composite reliability (CR)	Average variance extracted (AVE)
Trust in messages	0.87	0.93	0.65
Risk perception	0.87	0.87	0.58
Risk reduction preferences	0.89	0.89	0.46

## 4. Analysis and Discussion

The sample’s demographics are as follows. Of the 516 valid samples, there were 205 males and 309 females. The greatest portion of them ranged between 26 and 35 years old (180, 35%). There were 123 subjects younger than 25 years old (24%), and 109 subjects ranged between 36 to 45 years old (21.3%), with only few over 46 years old. A majority of our subjects had a college degree (53.9%) and the second largest proportion had a high school degree (26.6%) (Table 7).

**Table 7:** The demographics of samples.

Demographics	Classification	Number	Percentage (%)
Gender	Females	309	60.1
	Males	205	39.9
Age	Younger than 25	123	24
	25~35	180	35
	36~45	109	21.3
	46~55	60	11.7
	56 and older	41	8
Education	Less than high school degree	76	14.8
	High school degree	136	26.6
	College degree	276	53.9
	Master degree and over	24	4.7
Total	N = 516		

### 4.1. Difference Test of Consumer Attributes’ Effects on Risk Perception and Risk Reduction Preferences

*Gender.* To determine whether gender makes difference on risk perception and risk reduction preferences in avian influenza issues, we used an independent sample T test. The result is shown in Table 8. We found that gender makes no significant difference [45].

**Table 8:** T-test results of relationships between gender and risk perception and level of risk reduction preferences.

Variables	Female (n = 309)	Male (n = 205)	Mean deviation	t	*p*-Value
Risk perception	19.84	19.71	0.13	0.35	0.73
Level of risk	38.17	36.91	1.26	1.89	0.06
reduction preferences					

**Table 9:** ANOVA of relationships between age and risk perception and level of risk reduction preferences.

Variables	Sum of *Squares*	*df*	Mean Square	*F*	*p*-Value	Scheffe *Post hoc* Test
Risk perception	152.675	4	38.169	2.647	0.033 *	2,3,4 > 1
	7,311.004	507	14.420			
	7,463.680	511				
Level of risk reduction preferences	1,773.605	4	443.401	8.973	0.000 **	2,3,4 > 1,5
	25,053.137	507	49.414			
	26,826.742	511				

* *p* < 0.05; ** *p* < 0.01; Scheffe *Post hoc* Test: 1: Younger than 25; 2: 26~35 age; 3: 36~45 age; 4: 46~55 age; 5: 56 and older.

*Age.* To determine whether age makes a significant difference on risk perception and risk reduction preferences in avian influenza issues, we divided subjects into five age groups: younger than 25, 26 to 35, 36 to 45, 46 to 55, and older than 56. We analyzed the data with an ANOVA, and the results are shown in Table 9. We found that age had a significant effect. 

*Education.* To determine whether education makes significant difference on risk perception and risk reduction preferences in avian influenza issues, we divided subjects into four education-level groups: junior high school and below, high school, college, and master’s degree and beyond. We analyzed the data with an ANOVA, and the results are shown in Table 10. We found that education level had a significant effect.

**Table 10:** ANOVA of relationships between risk perception and level of risk reduction preferences to education.

Variables	Sum of *Squares*	*df*	Mean Square	*F*	*p*-Value	Scheffe *Post hoc* Test
Risk perception	123.503	3	41.168	2.844	0.037 *	3 > 1
	7,352.864	508	14.474			
	7,476.367	511				
Level of risk reduction preferences	988.994	3	329.665	6.476	0.000 **	2,3 > 1
	25,858.223	508	50.902			
	26,847.217	511				

* *p*< 0.05, ** *p* < 0.01; Scheffe *Post hoc* Test: 1: Less than high school degree; 2: High school degree; 3: College degree; 4: Master degree and over.

### 4.2. Correlation Analysis among Trust in Messages, Risk Perception, and Level of Risk Reduction Preferences

This research tested the three variables’ initial correlation through Pearson’s correlation. The result shown in Table 11 indicates that as the elements of trust in messages, both argument quality and source credibility have significant medium-lower positive correlation with risk perception and level of risk reduction preferences. This indicates that the more trust consumers have in the information about avian influenza, the higher their risk perception of avian influenza and the more likely they are to take actions to reduce risks. Although this demonstrated that trust is related to risk perception in the present study, we should note that most studies showed negative relationships between trust in messages and risk perception. Risk perception itself has significant medium-positive correlation with level of risk reduction preferences. This implies that the higher risk perception consumers have, the higher their level of risk reduction preferences. This correlation results on the positive correlation between risk perception and risk reduction strategies [5,42].

**Table 11:** Correlation analysis of relationships among trust in messages, risk perception, and level of risk reduction preferences.

	Trust in messages	Risk perception	Risk reduction preferences
Trust in messages	1.00	-	-
Risk perception	0.31 *	1.00	-
risk reduction preferences	0.43 *	0.46 *	1.00

* *p* < 0.05 (two-tailed).

### 4.3. The Fitness Analysis

This research used the Lisrel 8.7 software as the analysis tool for the model. The result of the Normed Fit Index (NFI) was 0.93, the Non-Normed Fit Index (NNFI) was 0.93 and the Comparative Fit Index (CFI) was 0.94. We could find that fit measures all achieved the desired value. 

### 4.4. Path Analytic Model

Path coefficients are standardized versions of linear regression weights which can be used in examining the possible causal linkage between statistical variables in the structural equation modeling approach [48]. Thus, path analysis was developed as a method of decomposing correlations into different pieces for interpretation of effects. Therefore, this study used the path analysis to explore path coefficients and the intermediate effect among the trust in message of avian influenza, risk perception and risk reduction preferences of the framework in this study.

We summed items’ scores for trust in messages, risk perception, and risk reduction preferences separately as representative scores of each variable. Then we used the path analysis and the SAS 8.0 statistics software as the analysis tool for the model. Because previous studies had found that consumers’ age and education related significantly to their avian influenza risk perception, we examined the relationships of age and education to risk perception, and we obtained results consistent with the previous findings. The age and education categories in this research refer specifically to consumers’ actual current age and their years of education as a continuous variable.

The results of the model analysis are shown in Table 12. Consumers’ age and education related significantly to the differences in their risk perception of avian influenza. Their path coefficients were 0.15 and 0.24, respectively, and reached a significant level. From this result, we conclude that both the consumers’ age and education exercised a significantly positive effect on their risk perception of avian influenza, with the elders and more highly educated consumers having a higher level of risk perception. In addition, the trust in avian influenza messages had a significant influence on the risk perception of avian influenza. Its path coefficient was 0.29 and reached a significant level, demonstrating that consumer trust in messages exercised a significantly positive effect on the risk perception of avian influenza. The results suggested that the consumers’ risk perception increases with an increase in their trust in messages. Thus, the analysis rejected our alternative hypothesis H1: Consumers’ trust in messages has no significant influence on their risk perception of avian influenza. Instead, it indicated that trust in messages had exercised a significant and positive effect on the risk perception of avian influenza.

**Table 12:** Path analysis of the model.

Model	X	Y	Path coefficient (t)	Model test ( *F*)
1	Age	Consumers’ risk perception	0.15 ** (3.00)	23.50 **
	Education	of avian influenza	0.24 ** (4.92)	
	Trust in messages		0.29 ** (6.90)	
2	Risk perception	risk reduction preferences	0.37 ** (9.06)	82.17 **

* *p* < 0.05, ** *p* *<* 0.01.

**Figure 2:**
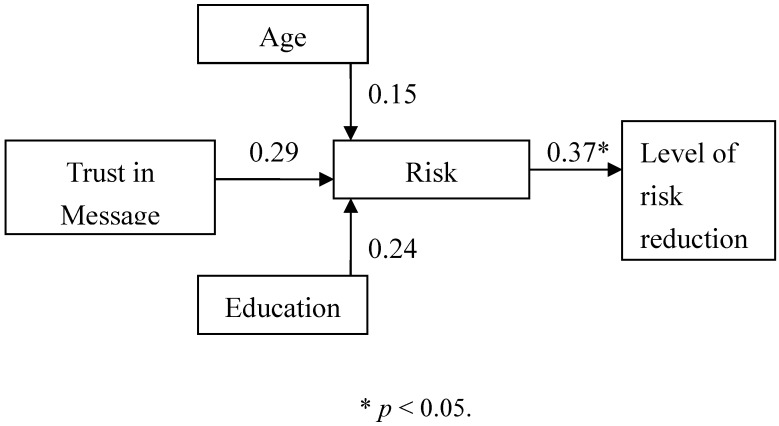
Path coefficient of model.

Consumers’ risk perception produced by avian influenza messages also has a significant effect on their level of risk reduction preferences. Its path coefficient was 0.37 and reached a significant level, demonstrating that consumers’ risk perception had exercised significant and positive effect on their level of risk reduction preferences.” Thus, the analysis also rejected H2: Consumers’ risk perception does not significantly influence their level of risk reduction preferences. Instead, it indicated that consumers’ risk perception of avian influenza had exercised a significant and positive effect on their level of risk reduction preferences, and the full model path coefficient is shown in Figure 2.

## 5. Conclusions and Suggestions

Due to the diversification of modern media communication today, the public receives news and information through various media channels. We have examined how people organize their perceptions and attitudes on receiving risk event messages, hoping that the results of this analysis will be available and useful to the government and risk management professionals. The paper developed the following conclusions.

### 5.1. Effect of Trust in Messages (Argument Quality and Source Credibility) on Risk Perception

In information processing, consumers’ consideration of information (weighing possible options) may change consumers’ *risk perception* and *risk reduction preferences* when they purchase products. This study found that consumers had a higher level of trust in messages and a higher risk perception of avian influenza after reading the avian influenza messages. Although most studies cited prove that trust can reduce people’s risk perception, consumers rely upon the expert sources of reports and the high-quality of messages, both of which promote consumers’ trust in messages, enable them to understand avian influenza and the concern about the development of an avian influenza epidemic, and thus increase consumers’ risk perception of avian influenza.

### 5.2. Effect of Risk Perception and Level of Risk Reduction Preferences

Consumers with higher risk perception have a higher level of risk reduction preferences and are more likely to take risk reduction strategies in the consumption of poultry. Therefore, it may encourage consumers to buy products with complete information and high-quality brands guaranteed by experts or the government.

### 5.3. Effect of Consumer Attributes on Risk Perception and Level of Risk Reduction Preferences

Risk perception and risk-reduction preference are not affected by gender or age group but by the level of education. To enhance risk perception and the level of risk reduction preferences, we suggest that the government should produce different levels of messages suitable for people of various education levels.

Based on the above results, consumers do not belong to the group which has High-Level consideration of avian influenza in theory only when avian influenza has spread or outbreak that makes consumers to have a higher risk perception of avian influenza. Therefore, it has two ways to elevate their risk perception of avian influenza. The first one is the source cue: identity and credibility of information sources, such as by government officials to release the message (identity status), or agriculture authority, experts and scholars (high confidence); through the credibility of the media or post the correct message on the official website by the government. The second one is the message cue: the study found that argument quality is the key point to affect the public thinking. The result shows that the more message of avian influenza consumers have, the more changes of attitude they have.

In fact, when avian influenza has already come, people will develop the strategy to reduce the risk perception, for example, consumers should try to buy meat products with the brand or qualifying mark. We suggest that for effective risk event management in the future, government units should produce various trusted and high-quality messages through experts and reliable media in formats and wording suitable for different segments of people. Such messaging can give people a clear impression of risk events and raise their risk perception, which can in turn increase their sense of security in using behavioral strategies to reduce the negative effects of risks and to promote the well-being of society. 
